# Factorial validation of the Attitudes toward Women Scale for Adolescents (AWSA) in assessing sexual behaviour patterns in Bolivian and Ecuadorian adolescents

**DOI:** 10.3402/gha.v7.23126

**Published:** 2014-01-23

**Authors:** Lina Jaruseviciene, Sara De Meyer, Peter Decat, Apolinaras Zaborskis, Olivier Degomme, Mildrett Rojas, Salazar Arnold Hagens, Nancy Auquilla, Bernardo Vega, Anna C. Gorter, Miguel Orozco, Jeffrey V. Lazarus

**Affiliations:** 1 Department of Family Medicine, Lithuanian University of Health Sciences, Kaunas, Lithuania; 2 International Centre for Reproductive Health, Ghent University, Ghent, Belgium; 3 South Group, Cochabamba, Bolivia; 4 University of Cuenca, Cuenca, Ecuador; 5 Instituto Centro Americano de Salud, Managua, Nicaragua; 6 Centro de Investigación y Estudios de la Salud, Managua, Nicaragua; 7 CHIP, WHO Collaborating Centre, Copenhagen University, Copenhagen, Denmark

**Keywords:** adolescents, gender attitudes, sexual behaviour, contraceptive use, Latin America

## Abstract

**Background:**

Adolescents’ health is greatly influenced by social determinants, including gender norms. Although research has shown that there is an association between gender attitudes and adolescents’ sexual behaviour, few studies have assessed this relationship carefully. The Attitudes toward Women Scale for Adolescents (AWSA) is widely used to assess gender attitudes among adolescents; however, to our knowledge it has not been applied in Latin America.

**Objective:**

To apply AWSA in Latin America for the first time, to perform a factorial validation of this scale and to assess the relationship of gender attitudes and sexual behaviour in Bolivian and Ecuadorian adolescents.

**Design:**

This cross-sectional study was carried out in 2011 among 14–18 year olds in 20 high schools in Cochabamba (Bolivia) and six in Cuenca (Ecuador) as a part of a larger project. Schools were purposively selected. A Spanish version of the 12-item AWSA was employed for this study. The assessed aspects of adolescent sexual behaviour were: reported sexual intercourse, reported positive experience during last sexual intercourse and reported current use of contraception. The psychometric properties of AWSA were investigated, and both explanatory and confirmatory factorial analyses were performed.

**Results:**

The number of questionnaires included in the analysis was 3,518 in Bolivia and 2,401 in Ecuador. A factorial analysis of AWSA resulted in three factors: power dimension (PD), equality dimension (ED) and behavioural dimension (BD). ED showed the highest correlates with adolescent sexual behaviour. Higher scores of this dimension were associated with a more positive experience of sexual relationships, a higher current use of modern contraception and greater sexual activity among girls.

**Conclusions:**

This study revealed a three-factorial structure of AWSA and demonstrated that by employing factors, the sensitivity of AWSA increases as compared to using the scale as a whole to assess sexual behaviour. This could have important implications for future research on gender and the sexual experiences of adolescents.

Adolescents’ health is greatly influenced by social determinants (1, 2), and gender norms are among the major social structural determinants that generate poorer health outcomes for both sexes (2). Since social norms and values related to gender are often internalized by adolescents before their sexual debut, their sexual attitudes and behaviour are likely to be shaped by these norms (3–5). Studies show that less egalitarian gender norms are associated with more risky sexual behaviour among adolescent males (3–7). For adolescent females, there is an association between less egalitarian gender norms and greater vulnerability to negative sexual and reproductive health outcomes (6, 8, 9).

Since internalized gender attitudes are related to the sexual behaviour of adolescents (10), research often aims to assess societal trends and developmental patterns in relation to gender attitudes. Studies clearly demonstrate differences in male and female gender attitudes and reveal a shift toward more egalitarian attitudes being adopted by younger generations in some countries (11–13). In spite of these societal changes, the discrepancy between males’ and females’ gender attitudes remains the greatest among adolescents (14, 15). Research on developmental patterns suggests that as cognitive abilities increase in late childhood, rigid gender perspectives formed in early childhood become more flexible; however, gender attitudes become less egalitarian again during adolescence (16, 17). Although research has demonstrated an association between gender attitudes and adolescents’ sexual behaviour (3, 8, 18), few studies have assessed this relationship systematically (5).

The Attitudes toward Women Scale (AWS) is the most widely used instrument to assess attitudes about women's right and roles in society (19). It was developed in 1972 as a 55-item self-reported instrument (20) and was subsequently reduced to 25, 21 and 15 items (20–22). Originally, the AWS scale placed individuals ‘on a continuum of attitudes ranging from traditional to egalitarian’ (20).

However, other studies raised concern about its one-dimensional structure, indicating that separate factor scores might be more appropriate than a total score (19).

The Attitudes toward Women Scale for Adolescents (AWSA), based on AWS, is widely used to assess gender attitudes among adolescents (16, 23–26). However, only Gibbons have performed a factor analysis of AWSA. Their study revealed three factors: traditional roles in family and work, personal freedom and time of sexual initiation for girls (27).

Researchers increasingly have explored the relationship between gender norms and sexual and reproductive health issues in Latin America (18, 28, 29). However, to our knowledge, AWSA has not been employed in researching adolescent sexual behaviour in Central or Latin America countries. The aims of this study were to use AWSA in this part of the world for the first time, to perform a factorial validation of this scale and to assess the relationship between gender attitudes and sexual behaviour in Bolivian and Ecuadorian adolescents.

## Methods

This paper reports on one component of Community-Embedded Reproductive Health Care for Adolescents in Latin America (CERCA), a European Commission-funded interventional research project (30). CERCA seeks to contribute to global knowledge about how primary health care can be more responsive to the sexual and reproductive health needs of adolescents. Its immediate objective is to create a community-based model to improve adolescent sexual and reproductive health in Bolivia, Ecuador and Nicaragua.

This paper reports on the results of a cross-sectional study performed in Cochabamba, Bolivia, and Cuenca, Ecuador, in 2011. Schools were purposively selected following a strategy developed by the CERCA consortium: 1) primary health care centres that were part of the intervention were selected: two in Cochabamba and three in Cuenca; 2) high schools that fell within the area of coverage of these health care centres were identified (12 in Cochabamba and three in Cuenca); and 3) control high schools were chosen in the area of coverage of other primary health care centres (eight in Cochabamba and three in Cuenca). The selected control schools had similar characteristics (socioeconomic indicators, geographic location and the size of school) to the intervention schools: mostly an urban environment, average income; however, there was a mix of income levels from the very poor to the very rich. The survey was performed in both intervention and control schools (20 out of a total of 1,100 schools in Cochabamba and six out of a total 127 schools in Cuenca) before the intervention started.

## Ethical issues

In both countries, the study was approved by ethical committees (Tribunal de Ética Medica, Colegio Medico de Cochabamba; Comisión de Bioética Facultad de Sciencias Médicas, Universidad de Cuenca). The applied procedures in the countries differed pending the national legislation. In Bolivia, the permission of the Ministry of Education and institutional permission of all selected schools was received. Subsequently, adolescents signed an informed consent form. In Ecuador, the institutional permission of selected schools was received; all parents or guardians of students from the selected schools were then informed about the study and given the option of refusing to allow their children to enrol. There were 11 parents who refused their children's participation in the survey. Only those whose parents did not refuse were asked to sign the informed consent form.

In both countries, all study participants were informed about the selection procedure, the purpose of the questionnaire and the planned publications. They were informed of their right to refuse to participate and the protection of collected data and guaranteed confidentiality.

### Participants

The survey was performed in 20 high schools in Bolivia (Cochabamba) and six in Ecuador (Cuenca).

At selected schools, interviewers visited all classrooms on the day of the survey. The interviewers – specially trained CERCA team staff – invited all 14–18-year-old students who were present (other than the 11 in Ecuador whose parents had refused) to complete the self-administered questionnaire after signing an informed consent form. No adolescents refused in Bolivia, while two adolescents refused in Ecuador.

### Measures

A native Spanish speaker translated the scale from English to Spanish; a native English speaker provided back translation. Neither translator was part of the CERCA team. The scale was pilot-tested before the survey among 38 adolescents in Cochabamba and 33 adolescents in Cuenca to check, among other things, the potential ambiguity and difficulty in understanding questions, the clarity of instructions, difficulty in responding to questions and the design of questionnaire. Minor language revisions to the scale were made after this pilot testing.

AWSA consists of 12 items, all of which were employed in this study (15):

V01 Swearing is worse for a girl than for a boy.

V02 On a date, the boy should be expected to pay all expenses.

V03 On average, girls are as smart as boys.

V04 More encouragement in a family should be given to sons than daughters to go to college.

V05 It is alright for a girl to want to play rough sports like football.

V06 In general, the father should have greater authority than the mother in making family decisions.

V07 It is alright for a girl to ask a boy out on a date.

V08 It is more important for boys than girls to do well in school.

V09 If both husband and wife have jobs, the husband should do a share of the housework such as washing dishes and doing the laundry.

V10 Boys are better leaders than girls.

V11 Girls should be more concerned with becoming good wives and mothers than desiring a professional or business career.

V12 Girls should have the same freedom as boys.

For each item, respondents were asked to indicate their level of agreement or disagreement on a Likert-type scale ranging from 1 (‘strongly agree’) to 4 (‘strongly disagree’). Items 3, 5, 7, 9 and 12 were reverse-scored. All scores were subsequently summed and divided by 12, producing a total score of attitudes toward women. A higher score indicated a less traditional attitude while a lower score indicated a more traditional attitude.

Three variables related to sexual activity were measured: reported sexual intercourse, reported positive experience during last sexual intercourse and reported current use of contraception. Adolescents who indicated that they had ever had sex were attributed to the group that reported sexual intercourse. Reported positive experience during last intercourse was measured with a single item: ‘How did you feel the last time you had sexual intercourse?’ Only those who selected the response, ‘It was a special experience’, were attributed to the group with a positive sexual experience. Study participants who selected other answers (‘It was not a special experience’, ‘It was a bad experience’, ‘Don't know’, or ‘Others’) constituted the reference group. The variable ‘Current use of contraception’ reflected reported adolescent use of oral contraceptives, intrauterine devices, injections, implants and condoms (if used three times during last three episodes of sexual intercourse).

### Statistical analysis

SPSS 21.0 supplemented with AMOS was used for the analyses. In order to compare results between countries, the analysis controlled for the effect of gender and age to avoid discrepancies in the sampling frame between countries. For this reason, crude estimates of group frequencies were weighted according to the average number of respondents by sex and age within each country. A negligible change in the total count of subjects was observed due to weighting (Table 1). Descriptive statistics were employed to calculate means and standard deviations of the quantitative continuous variables, as well as to calculate percentages of the categorical data.

**Table 1:** Crude and weighted samples of respondents from Bolivia and Ecuador

		Crude sample			Weighted sample		
			
		Bolivia	Ecuador	Total	Bolivia	Ecuador	Total
Age	14	518	656	1,174	705	481	1,186
	15	803	651	1,454	704	482	1,186
	16	877	580	1,457	705	477	1,182
	17	877	398	1,275	705	479	1,184
	18	443	116	559	704	489	1,193
Total		3,518	2,401	5,919	3,523	2,408	5,931

A two-sided *t*-test for equality of means was used to determine whether the measures of the scale differed between boys and girls as well as between Bolivian and Ecuadorian adolescents. Associations between dichotomised measures of the scale and different forms of sexual behaviour were estimated using odds ratios (OR) with 95% confidence intervals (CI) in a binary logistic regression analysis.

The evaluation of the distribution of scores for each item and for the total has been used to analyse the feasibility of measures with scales (31, 32). A score would generally be considered acceptable if the values were distributed in a normal curve, or at least symmetrically. When the distribution of the scale was skewed, the floor and ceiling effects were considered. In this study, we determined these effects by examining the deviance in distribution symmetry, e.g. 50% less proportion of subjects who ‘agree strongly’ or ‘agree’ with the statement (negative values of this measure indicate a floor effect while positive values indicate a ceiling effect). The presence of floor and ceiling effects was defined as 15% or more of deviance in distribution symmetry.

In order to understand the interrelations among the full set of measures and to confirm the inherent structure of the scale, an explanatory factorial analysis (EFA) was performed. Using the SPSS ‘Factor analysis’ procedure, we carried out a Principal Component Factor analysis with a Varimax Rotation. The appropriateness of these factor models was evaluated with Bartlett's test of sphericity and the Kaiser-Meyer-Olkin (KMO) measure of sampling adequacy (*p*<0.001 and KMO> 0.5, if the sample is adequate). Factors were extracted based on the break point of successive eigenvalues (>1) identified in a Scree Plot, item factor loadings (>0.4) and interpretability. Next, in EFA, the factor scores for each respondent were calculated and saved for further analysis. In addition, these measures were dichotomised into negative and positive values (with a zero cut-off point). Dichotomised values were used to assess the external (predictive) validity of the AWSA scale. Finally, we introduced confirmatory factor analysis (CFA) to confirm findings of the EFA and to elucidate the internal structure of the scale in different sub-samples of respondents. Indices of good fit such as the Comparative Fit Index (CFI), Tucker-Lewis Fit Index (TLI) and the Root Mean Square Error of Approximation (RMSEA) were assessed (adequate cut-off levels for model fit were set at >0.95 for the TLI and CFI and <0.08, respectively, for the RMSEA) (33). Both the EFA and CFA were performed on the entire sample as well as on the following sub-samples: Bolivian boys and girls together; Ecuadorian boys and girls together; Bolivian and Ecuadorian boys together; Bolivian and Ecuadorian girls together.

The Cronbach α was used as a measure of internal consistency of the total scale and its subscales. Each of the subscales was subjected to the Spearman-Brown prediction formula to adjust its reliability to the reliability of the full 12-item test (34). A Cronbach α ≥0.70 was considered acceptable. Furthermore, item-total correlations (Pearson) were investigated.

Three external criteria were used to assess the external (predictive) validity of the AWSA scale in our study: reported sexual intercourse; reported positive experience during last sexual intercourse; and reported current use of contraceptives.

## Results

### Respondents and feasibility of AWSA

A total of 3,519 questionnaires were collected in Bolivia and 2,403 in Ecuador. As one questionnaire in Bolivia and two questionnaires in Ecuador were incomplete, the number of questionnaires included in the analysis was 3,518 in Bolivia and 2,401 in Ecuador. Half (50%) of the respondents in the total sample were male (50.1% in Bolivia and 49.8% in Ecuador). The age structure of the national and total samples is presented in Table 1. More than two-thirds of the total sample (70.7%) consisted of adolescents aged 15–17 years (72.7% in Bolivia and 67.8% in Ecuador). Adolescents aged 14 years formed almost one-fifth of the total sample (19.8%), while less than one-tenth of the sample (9.4%) was aged 18 years.

The overall sample completed 93.9% of the items in the survey (94.1% in Bolivia and 93.6% in Ecuador).

Table 2 shows the distribution of scores by AWSA item. Notable floor effects were detected for items V01, V03, V09 and V12. Notable ceiling effects were detected for item V11. It was not likely that the floor or ceiling effects were more prominent for the items that were reverse-coded.

**Table 2:** Distribution of scores in response to items on the Attitudes toward Women Scale for Adolescents (AWSA)

AWSA item	Strongly agree (=1)	Agree (=2)	Disagree (=3)	Strongly disagree (=4)	Assessment of distribution symmetry^a^
V01 Swearing is worse for a girl than for a boy	2,850 (48.5%)	1,938 (33.3%)	684 (11.6%)	404 (6.9%)	−31.8%
V02 On a date, the boy should be expected to pay all expenses	1,763 (30.0%)	1,964 (33.4%)	1,737 (29.5%)	415 (7.1%)	−13.4%
V03 On average, girls are as smart as boys^b^	2,486 (42.3%)	2,126 (36.2%)	988 (16.8%)	275 (4.7%)	−28.5%
V04 More encouragement in a family should be given to sons than daughters to go to college	1,025 (17.5%)	1,620 (27.6%)	2,043 (34.8%)	1,180 (20.1)	+4.9%
V05 It is alright for a girl to want to play rough sports like football^b^	1,574 (26.7%)	2,432 (41.3%)	1,373 (23.3%)	511 (8.7%)	−18.0%
V06 In general, the father should have greater authority than the mother in making family decisions	576 (9.8%)	944 (16.1%)	2,798 (47.6%)	1,555 (26.5%)	+24.1%
V07 It is alright for a girl to ask a boy out on a date^b^	1,091 (18.6%)	2,196 (37.4%)	1,636 (27.9)	952 (16.2)	−6.0%
V08 It is more important for boys than girls to do well in school	974 (16.6%)	1,599 (27.2%)	2,382 (40.6%)	914 (15.6%)	+6.2%
V09 If both husband and wife have jobs, the husband should do a share of the housework such as washing dishes and doing the laundry^b^	3,109 (52.9%)	2,366 (40.2%)	262 (4.5%)	148 (2.5%)	−43.1%
V10 Boys are better leaders than girls	634 (10.8%)	1,154 (19.7%)	2,724 (46.5%)	1,350 (23.0%)	+19.5%
V11 Girls should be more concerned with becoming good wives and mothers than desiring a professional or business career	586 (10.0%)	731 (12.4%)	2,327 (39.6%)	2,229 (38.0%)	+27.6%
V12 Girls should have the same freedom as boys^b^	2,560 (43.5%)	2,194 (37.2)	858 (14.6%)	279 (4.6%)	−30.7%

a ‘−’ floor effect, and ‘ + ' ceiling effect, calculated as 50% – (proportion of ‘agree strongly’ and ‘agree’).

b Reverse-scored.

### Explanatory factorial analysis

The explanatory factorial analysis (EFA) on the total study sample revealed a three-factor solution, which accounted for 44.8% of total variance. Table 3 presents the factors structure of the AWSA (the result of the Principal Component Analysis [PCA] with Varimax normalized rotation is reported). Factorial analysis on sub-samples of Bolivian and Ecuadorian adolescents (with males and females analysed separately as well as together) supported the three-factor solution and confirmed the factor structure (results not presented). The appropriateness of these factor models was evaluated by Bartlett's test of sphericity and KMO measure of sampling adequacy.

**Table 3:** Factor loadings of the principle components analysis and item-total correlation of the Attitudes toward Women Scale for Adolescents (AWSA)

	Loadings				
		
	Three factors analysis				
			
AWSA item^a^	F1 (PD)	F2 (ED)	F3 (BD)	Single factor analysis	Item-total correlation
V10 Boys are better leaders than girls	**0.73**	0.19	−0.09	0.73	0.60
V08 It is more important for boys than girls to do well in school	**0.67**	−0.02	0.13	0.62	0.54
V06 In general, the father should have greater authority than the mother in making family decisions	**0.64**	0.13	0.11	0.65	0.56
V11 Girls should be more concerned with becoming good wives and mothers than desiring a professional or business career	**0.63**	0.16	−0.05	0.63	0.55
V04 More encouragement in a family should be given to sons than daughters to go to college	**0.58**	0.03	0.35	0.59	0.56
V12 Girls should have the same freedom as boys^b^	0.11	**0.63**	−0.02	0.35	0.40
V09 If both husband and wife have jobs, the husband should do a share of the housework such as washing dishes and doing the laundry^b^	0.14	**0.61**	−0.20	0.34	0.35
V05 It is alright for a girl to want to play rough sports like football^b^	0.06	**0.60**	0.29	0.34	0.44
V03 On average, girls are as smart as boys^b^	0.12	**0.51**	−0.30	0.27	0.32
V07 It is alright for a girl to ask a boy out on a date^b^	−0.32	0.37	**0.64**	−0.05	0.24
V02 On a date, the boy should be expected to pay all expenses	0.21	−0.20	**0.60**	0.20	0.32
V01 Swearing is worse for a girl than for a boy	0.15	−0.09	**0.47**	0.17	0.30
Eigen value	2.35	1.66	1.37	2.57	
% of variance	19.6	13.8	11.4	21.5	
Total variance explained	44.8			21.5	
KMO measure of sampling adequacy	0.76				
Bartlett's test of Sphericity	<0.001				
Cronbach α: Total	0.71	0.48	0.24	0.61	
Boys	0.70	0.48	0.21	0.61	
Girls	0.66	0.43	0.33	0.58	
Adjusted Cronbach α^c^: Total	0.85	0.73	0.60	0.61	
Boys	0.85	0.73	0.52	0.61	
Girls	0.82	0.70	0.66	0.58	

a Items are sorted by loadings; factor rotation converged in eight iterations.

b Reverse-scored.

c Adjusted by Spearman-Brown prediction formula to 12 items scale.

F1 (PD) – power dimension; F2 (ED) – equality dimension; F3 (BD) – behavioural dimension. The bolded terms indicate the main loadings for corresponding dimensions.

The estimated loadings indicated that the first component combined five items. The maximal loading (0.73) was found for item V10, ‘Boys are better leaders than girls’. The remaining four items (V08, V06, V11 and V04) also had great loadings of the first component but relatively small loadings of the other two components. Higher values of the responses correspond to stronger disagreement with the item statements and thus attribute a higher level of power to girls and women. We interpreted this group of items as a factor of ‘power dimension’ (Factor PD).

The second component combined four items (V12, V09, V05 and V03, listed by weight) clearly distinguished by large loadings of the second component. Agreement with these statements indicated an attitude of striving for greater gender equality. This component was identified as a factor of ‘equality dimension’ (Factor ED).

Finally, the third component combined three items (V07, V02 and V01), which accounted for 11.4% of total variance. This component was identified as a factor of ‘behaviour dimension’ (Factor BD). As Table 3 indicates, the items of Factor PD had the greatest loadings, while items of Factor BD had the smallest loadings.

When Pearson correlation coefficients were calculated for the total score and the factorial estimations, there was a strong correlation (0.74) between the total score and Factor PD. Correlations between the total score and Factor ED and Factor BD were much weaker (0.54 and 0.41, respectively; all correlations were significant at *p*<0.01.)

### Internal consistency

Although various items of the AWSA were significantly correlated with each other, the internal consistency (Cronbach α) of the total scale for the entire sample was 0.61 (boys, 0.61; girls, 0.58; Bolivian respondents, 0.62; Ecuadorian respondents, 0.60).

The greatest inter-item correlation coefficients were obtained for the five items of Factor PD; coefficients ranged from 0.27 to 0.39. The corresponding Cronbach α was 0.71 (boys, 0.70; girls, 0.66; Bolivian respondents, 0.70; Ecuadorian respondents, 0.72), indicating an acceptable level of internal consistency of the subscale. The equal-length Spearman-Brown test produced a reliability coefficient of 0.85 (good internal consistency). Items in the scale consistently show high (>0.5) item-total correlations. The reliability of the individual items ranged from 0.66 to 0.71 for this subscale.

The inter-item correlations for the four items that form Factor ED ranged from 0.14 to 0.25 and the item analysis yielded Pearson reliability coefficients ranging from 0.62 to 0.65. The Cronbach α was 0.48 (boys, 0.48; girls, 0.43; Bolivian respondents, 0.49; Ecuadorian respondents, 0.43). The equal-length Spearman-Brown test produced a reliability coefficient of 0.73 (an acceptable level of internal consistency).

Items V01, V02 and V07 contributed little to the total scale (on average, the correlation between these items was 0.10). Due to that problem and to the small number of items, the Cronbach α for Factor BD was 0.24 (boys, 0.21; girls, 0.22; Bolivian respondents, 0.26; Ecuadorian respondents, 0.22). However, the equal-length Spearman-Brown test produced a reliability coefficient of 0.60.

Table 4 presents comparisons of the descriptive statistics for the AWSA between groups of respondents by country and gender. Comparisons between boys and girls were statistically significant, indicating a less traditional attitude among girls for the total scale (total score and one-factor solution) and for the items of Factor PD, but a more traditional attitude for the items of Factor BD. There was a slight difference between boys’ and girls’ scores for the items of Factor ED. Differences in responses from Bolivia and Ecuador were not statistically significant except for Factor ED, which showed slightly higher scores for the corresponding items among Ecuadorians.

**Table 4:** Descriptive statistics for the estimated measures of the Attitudes toward Women Scale for Adolescents (AWSA) by country and gender

Factor	Country	Gender	n	Mean±SE	Proportion of positive values (%)	*p* [F test]
F1 (PD)	Bolivia + Ecuador	Boys	2,964	−0.413±0.018	32.5	<0.001
		Girls	2,967	0.413±0.016	69.8	[F(1;5929) = 1216.8]
		Boys + girls	5,931	0±0.013	51.2	
	Bolivia	Boys	1,765	−0.410±0.022	32.2	<0.001
		Girls	1,758	0.427±0.020	71.2	[F(1;3521) = 770.4]
		Boys + girls	3,523	0.008±0.017	51.7	
	Ecuador	Boys	1,200	−0.416±0.029	33.0	<0.001
		Girls	1,209	0.391±0.025	67.7	[F(1;2406) = 450.4]
		Boys + girls	2,408	−0.011±0.021^a^	50.4	
F2 (ED)	Bolivia + Ecuador	Boys	2,964	−0.070±0.019	46.5	<0.001
		Girls	2,967	0.070±0.017	53.5	[F(1;5929) = 29.6]
		Boys + girls	5,931	0±0.013	50.0	
	Bolivia	Boys	1,765	−0.182±0.025	42.4	<0.001
		Girls	1,758	0.053±0.022	53.0	[F(1;3521) = 49.6]
		Boys + girls	3,523	−0.065±0.017	47.7	
	Ecuador	Boys	1,200	0.094±0.029	52.5	0.969
		Girls	1,209	0.095±0.028	54.3	[F(1;2406) = 0.1]
		Boys + girls	2,408	0.095±0.020^b^	53.4	
F3 (BD)	Bolivia + Ecuador	Boys	2,964	0.196±0.018	60.6	<0.001
		Girls	2,967	−0.195±0.018	43.3	[F(1;5929) = 235.8]
		Boys + girls	5,931	0±0.013	51.9	
	Bolivia	Boys	1,765	0.181±0.023	60.7	<0.001
		Girls	1,758	−0.166±0.024	45.1	[F(1;3521) = 109.6]
		Boys + girls	3,523	0.008±0.017	52.9	
	Ecuador	Boys	1,200	0.217±0.027	60.4	<0.001
		Girls	1,209	−0.239±0.029	40.9	[F(1;2406) = 131.1]
		Boys + girls	2,408	−0.012±0.020^c^	50.6	
One-factor solution	Bolivia + Ecuador	Boys	2,964	−0.373±0.018	33.6	<0.001
		Girls	2,967	0.373±0.016	65.7	[F(1;5929) = 959.8]
		Boys + girls	5,931	0±0.013	51.9	
	Bolivia	Boys	1,765	−0.417±0.023	31.5	<0.001
		Girls	1,758	0.384±0.021	66.6	[F(1;3520) = 658.4]
		Boys + girls	3,523	−0.017±0.017	49.0	
	Ecuador	Boys	1,200	−0.308±0.029	36.7	<0.001
		Girls	1,209	0.357±0.025	64.4	[F(1;2406) = 310.1]
		Boys + girls	2,408	0.026±0.020^d^	50.6	
Total score	Bolivia + Ecuador	Boys	2,786	2.656±0.007	34.0#	<0.001
		Girls	2,783	2.863±0.007	54.2	[F(1;5566) = 417.5]
		Boys + girls	5,569	2.759±0.005	44.1	
	Bolivia	Boys	1,653	2.635±0.010	32.3	<0.001
		Girls	1,662	2.869±0.009	55.3	[F(1;3313) = 314.9]
		Boys + girls	3,315	2.752±0.007	43.8	
	Ecuador	Boys	1,132	2.686±0.012	36.5	<0.001
		Girls	1,121	2.854±0.011	52.7	[F(1;2251) = 112.9]
		Boys + girls	2,254	2.770±0.008^e^	44.5	

a *p*=0.473 [F(15,929) = 0.5]

b *p*<0.001 [F(15,929) = 36.6]

c *p*=0.448 [F(15,929) = 0.6]

d *p*=0.101 [F(15,929) = 2.7]

e *p*=0.108 [F(15,566) = 2.6] comparing adolescents from Bolivia and Ecuador.

# Here and below, proportion of cases with total score >2.75.

### Confirmatory factor analysis

The dimensionality of the AWSA scale was assessed using confirmatory factor analysis (CFA). Based on the above EFA, we hypothesized that CFA would show three factors. In addition, we postulated that common factors may be correlated, because each of them has a connection with a limited number of scale items (in contrast to EFA, which used PCA, common factors are connected with all scale items).

**Fig. 1:**
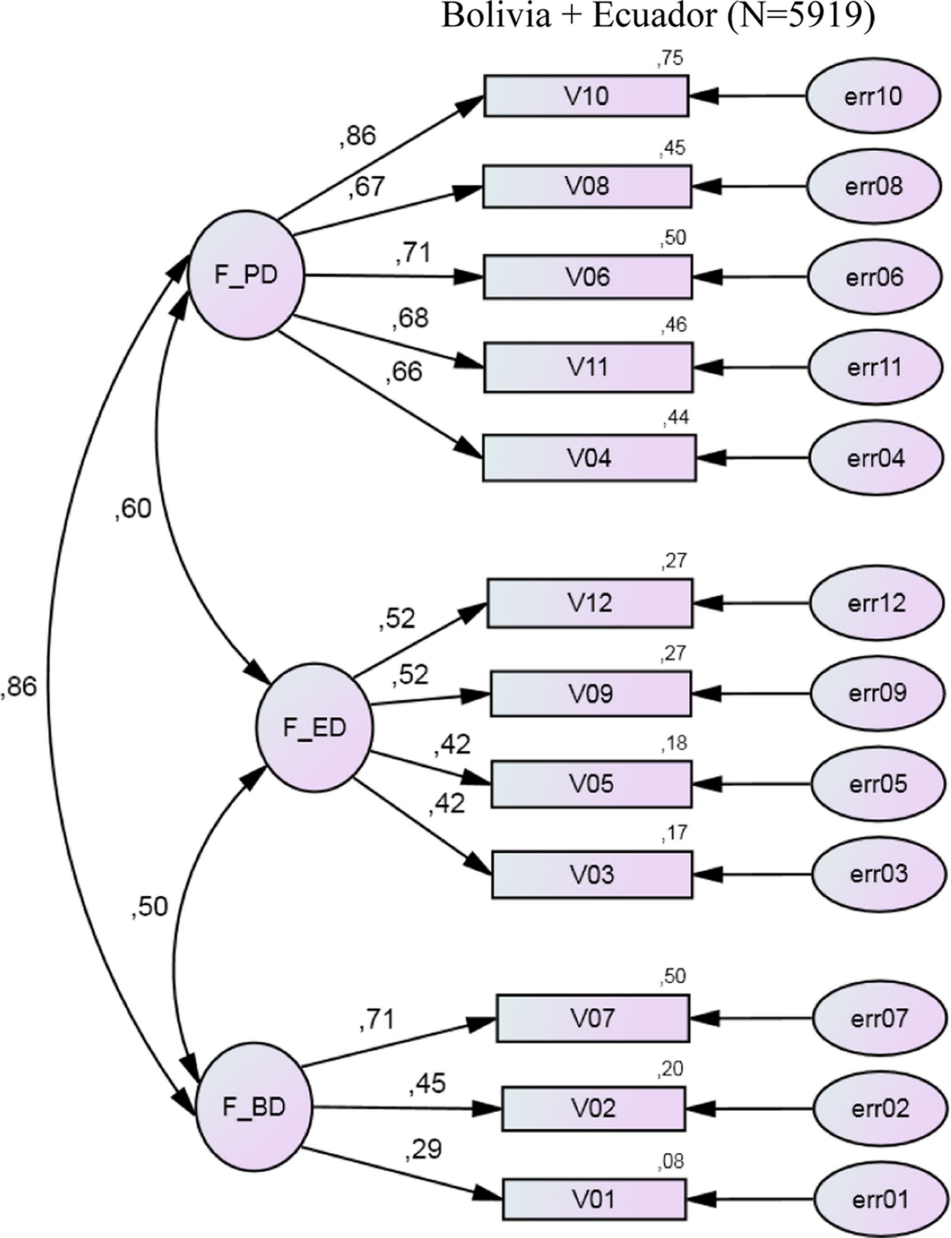
Path diagram of confirmatory factor analysis with standardized estimates (data from Bolivia and Ecuador, N = 5,919). V01÷V12 are observed variables (AWSA items); err01÷err12 are unobserved variables; F_PD is power dimension, F_ED is equity dimension and F_BD is behavioural dimension.

Figure 1 demonstrates a path diagram of CFA with standardized estimates, which was constructed on the basis of data from Bolivia and Ecuador. Factor loadings (the path coefficients leading from the common factors to the observed variables) were found to be adequate, except for the path from the Factor BP to V01 – ‘Swearing is worse for a girl than for a boy’. The factors were moderately correlated (r=0.50÷0.86).

In order to compare the factor structure of AWSA between Bolivian and Ecuadorian adolescents, we ran a CFA and tested whether both countries followed the same factor structure. These analyses confirmed the *a priori* expectations (Table 5). Indices of good fit were assessed. A RMSEA score (0.071<0.08; 90% CI: 0.067–0.076) indicates good model fit in the total sample, as well as in both the Bolivian and Ecuadorian samples. Therefore, other fit indices, namely CFI and TLI, suggested insufficient (<0.95) model fit in all three datasets.

**Table 5:** Estimates of three-factor model of the Attitudes toward Women Scale for Adolescents (AWSA) scale obtained from confirmatory factor analysis of data from Bolivia and Ecuador

			Estimates		
			
			Bolivia+Ecuador	Bolivia	Ecuador
Standardized regression weights (factor loadings):					
V10	<---	F_PD	0.864	0.867	0.858
V08	<---	F_PD	0.669	0.653	0.686
V06	<---	F_PD	0.711	0.705	0.722
V11	<---	F_PD	0.679	0.697	0.651
V04	<---	F_PD	0.665	0.652	0.682
V12	<---	F_ED	0.522	0.569	0.459
V09	<---	F_ED	0.519	0.547	0.469
V05	<---	F_ED	0.422	0.374	0.588
V03	<---	F_ED	0.416	0.429	0.380
V07	<---	F_BD	0.709	0.737	0.710
V02	<---	F_BD	0.447	0.535	0.209
V01	<---	F_BD	0.289	0.298	0.174
Correlations:					
F_PD	<-- >	F_ED	0.604	0.608	0.676
F_PD	<-- >	F_BD	0.862	0.841	0.863
F_ED	<-- >	F_BD	0.501	0.404	0.825
Model fit:					
RMSEA (90% CI)			0.071 (0.067–0.076)	0.076 (0.074–0.079)	0.079 (0.076–0.082)
TLI			0.192	0.239	0.146
CFI			0.364	0.401	0.327

### External (predictive) validity

Table 6 demonstrates the external validity of the components of the AWSA scale. Although all three factors were associated with different aspects of adolescent sexual behaviour, Factor ED emerged as the dimension of gender attitudes that correlated most consistently with adolescent sexual behaviour. For example, Factor ED was the only factor that demonstrated an association with girls’ sexual activity in the total sample (Bolivia and Ecuador). Moreover, sexually active boys and girls with higher Factor ED scores were significantly more likely to report having a positive experience during last sexual intercourse (OR 1.81 for boys and 1.52 for girls). Higher Factor ED was also significantly associated with current contraception use by both boys and girls (OR 1.82 and 1.44, respectively).

**Table 6:** Association of gender scale factors with sexual intercourse, positive experience during last sexual intercourse and current use of contraception among boys and girls in Bolivia and Ecuador

		Bolivia		Ecuador		Bolivia + Ecuador	
				
	Value of the factor	Boys	Girls	Boys	Girls	Boys	Girls
Reported sexual intercourse							
F1 (PD)	Negative	35.9	17.2	31.5	22.3	34.1	19.5
	Positive	35.3	14.9	29.5	24.3	33.0	18.6
	OR (95% CI)	0.98 (0.79–1.20)	0.84 (0.68–1.21)	0.91 (0.70–1.19)	1.12 (0.84–1.49)	0.95 (0.81–1.12)	0.95 (0.78–1.16)
F2 (ED)	Negative	36.1	14.4	30.4	20.8	34.1	17.0
	Positive	35.2	16.6	31.3	25.9	33.5	20.5
	OR (95% CI)	0.96 (0.79–1.17)	1.18 (0.91–1.53)	1.04 (0.82–1.33)	**1.33 (1.02**–**1.74**)	0.97 (0.84–1.14)	**1.26 (1.04**–**1.51)**
F3 (BD)	Negative	27.6	15.6	28.7	23.0	34.1	19.5
	Positive	41.0	15.5	32.4	24.5	33.0	18.6
	OR (95% CI)	**1.83 (1.49**–**2.25)**	0.99 (0.76–1.29)	1.19 (0.92–1.53)	1.09 (0.83–1.53)	0.95 (0.81–1.12)	0.95 (0.78–1.16)
One-factor solution	Negative	35.8	15.7	31.0	21.1	33.9	18.0
	Positive	35.5	15.5	30.7	25.1	33.4	19.7
	OR (95% CI)	0.99 (0.80–1.22)	0.99 (0.75–1.30)	0.99 (0.77–1.27)	1.25 (0.94–1.66)	0.97 (0.83–1.15)	1.09 (0.90–1.33)
Total score	≤2.75	35.1	16.3	31.3	20.9	33.6	18.2
	>2.75	37.1	15.7	30.3	26.4	34.1	19.9
	OR (95% CI)	1.09 (0.88–1.36)	0.96 (0.74–1.25)	0.95 (0.73–1.24)	**1.36 (1.03–1.79)**	1.03 (0.87–1.21)	1.12 (0.92–1.35)
Reported positive experience during last sexual intercourse							
F1 (PD)	Negative	52.1	61.6	66.8	79.3	57.8	70.5
	Positive	63.2	59.8	68.4	73.3	65.5	66.8
	OR (95% CI)	**1.58 (1.11**–**2.25)**	0.93 (0.55–1.57)	1.07 (0.67–1.72)	0.72 (0.39–1.32)	**1.38 (1.04**–**1.83)**	0.84 (0.57–1.25)
F2 (ED)	Negative	52.3	51.3	62.1	69.2	55.5	60.5
	Positive	60.6	67.3	71.9	78.8	65.5	73.5
	OR (95% CI)	**1.41 (1.01**–**1.96)**	**1.96 (1.18**–**3.24)**	**1.56 (1.01**–**2.42)**	1.66 (0.96–2.88)	**1.52 (1.17**–**1.87)**	**1.81 (1.27**–**2.62)**
F3 (BD)	Negative	56.6	60.1	71.5	74.4	63.0	67.7
	Positive	55.3	60.7	64.9	76.0	58.8	68.5
	OR (95% CI)	0.95 (0.67–1.36)	1.02 (0.62–1.69)	0.74 (0.46–1.18)	1.09 (0.63–1.90)	0.84 (0.64–1.11)	1.04 (0.72–1.50)
One-factor solution	Negative	52.0	58.4	65.7	71.4	57.1	65.0
	Positive	63.9	61.4	70.2	76.9	66.6	69.6
	OR (95% CI)	**1.63 (1.14**–**2.34)**	1.13 (0.67–1.91)	1.23 (0.78–1.96)	1.33 (0.75–2.35)	**1.50 (1.13**–**1.99)**	1.23 (0.84–1.80)
Total score	≤2.75	53.6	55.6	65.9	62.7	58.3	58.6
	>2.75	61.4	63.7	69.1	80.6	64.5	72.9
	OR (95% CI)	1.38 (0.96–1.98)	1.40 (0.85–2.33)	1.16 (0.72–1.86)	**2.49 (1.42**–**4.39)**	1.30 (0.98–1.73)	**1.89 (1.30**–**2.75)**
Reported current use of contraceptives							
F1 (PD)	Negative	29.8	28.5	35.3	40.5	31.1	39.9
	Positive	29.3	30.9	33.3	29.9	31.8	29.4
	OR (95% CI)	0.97 (0.69–1.37)	1.12 (0.67–1.89)	0.92 (0.60–1.42)	0.63 (0.38–1.02)	1.03 (0.77–1.37)	**0.63 (0.43**–**0.91)**
F2 (ED)	Negative	24.6	24.8	30.4	27.7	27.7	25.2
	Positive	35.2	33.8	37.4	43.2	35.5	38.0
	OR (95% CI)	**1.66 (1.17**–**2.35)**	1.55 (0.91–2.63)	1.37 (0.88–2.11)	**1.99 (1.21**–**3.25)**	**1.44 (1.10**–**1.89)**	**1.82 (1.25**–**2.63)**
F3 (BD)	Negative	26.2	29.5	33.7	40.3	28.3	31.1
	Positive	31.6	29.8	35.2	31.1	32.8	34.8
	OR (95% CI)	1.30 (0.91–1.87)	1.101 (0.60–1.71)	1.07 (0.69–1.65)	0.67 (0.41–1.09)	1.24 (0.92–1.65)	1.18 (0.83–1.69)
One-factor solution	Negative	27.7	29.3	34.6	37.4	67.4	23.5
	Positive	33.5	29.8	33.3	34.9	60.8	28.3
	OR (95% CI)	1.31 (0.91–1.89)	1.02 (0.59–1.78)	0.94 (0.60–1.48)	0.90 (0.54–1.51)	**0.75 (0.57**–**0.99)**	1.29 (0.86–1.94)
Total score	≤2.75	28.6	28.9	32.6	27.0	30.1	28.0
	>2.75	33.3	31.3	32.8	39.1	32.9	35.3
	OR (95% CI)	1.25 (0.86–1.80)	1.18 (0.66–1.89)	1.01 (0.63–1.61)	**1.73 (1.02**–**2.94)**	1.14 (0.85–1.52)	1.40 (0.97–2.04)

Bolivia + Ecuador (N = 5919). The bolded terms are significant at p<0.05.

## Discussion

This study tested AWSA among Bolivian and Ecuadorian adolescents by investigating the psychometric properties of this instrument. In addition, it sought to reveal the factorial structure of the AWSA and to assess the external validity of AWSA components by measuring their relationship to the sexual behaviour of Bolivian and Ecuadorian adolescents.

The Cronbach α of the total scale for the entire sample, 0.61, was lower than that found in other studies: 0.71 (16) or 0.67 (25). Spence and Hahn (13) emphasized the need to assess the ceiling effects monitoring the utility of this instrument. Some AWSA items (e.g. V1, V3, V9) contributed more to the ceiling effects of the total scale. In future, these parameters should be regularly verified.

In our study, the means of the total score were higher for girls than for boys. More egalitarian gender attitudes among females were also demonstrated in previous investigations (24).

Given that the gender-role attitudes inventory is normally used to calculate the total score, Spence and Hahn (13) emphasized the importance of a single-factorial structure of the AWS inventory, on which basis AWSA was created. Contrary to the vision of Spence, who adheres to the single-factorial structure of the AWS inventory, our analysis of Bolivian and Ecuadorian adolescents’ gender attitudes adduces an argument in support of a three-factorial structure of the AWSA scale. This corresponds with the recent findings of Coello and Fernandez (35), who tested the 15-item AWS among Spanish adolescents. This study disclosed the multidimensionality of AWSA although psychometric parameters of these dimensions were not satisfactory. Byrne et al. (19) found that the factorial structure of the 20-item AWS scale was different across age groups.

Our study suggests the multifactorial nature of the AWSA, as was demonstrated earlier with the AWS inventory (19). More than two decades ago, the future utility of AWS was questioned in the context of increasing gender equality and changing attitudes about gender roles (36). Our study points out that the potential loss of reliability of a single-factorial structure of the AWSA inventory could indicate the changing trends of gender attitudes. Although this finding needs to be confirmed by additional research in different contexts, our data suggest the different social dynamics between the internal components of gender attitudes. The smallest difference among female and male attitudes on Factor ED – the equality dimension – could indicate increased general acceptance of egalitarian gender roles. Pronounced discrepancies among boys and girls for Factor PD – the power dimension – and Factor BD – the behavioural dimension – could indicate higher male support for expressions of behavioural equality and resistance to a more even distribution of power between men and women. In contrast, females are less supportive of Factor BD, but very ‘pro’ Factor PD.

The relationship between revealed factors and different aspects of sexual experience shows the external validity of these dimensions. The use of the total AWSA score or the one-factor solution could mask relationships between gender attitudes and components of sexual behaviour. In our study, there was a strong correlation between the total score and the estimation obtained from the one-factor analysis and F1 (PD) obtained from the three-factor analysis. However, the correlation of Factors ED and Factors BD with the total score was much weaker, indicating the specificity of these factorial measures. External validation confirmed this insight: among all three revealed factors, only Factor ED seems to have a consistent relationship with reported sexual activity, reported positive experience during last sexual intercourse and reported current use of contraception. This finding is in line with Loo's (37) insight that a subscale score of the large AWS version is better than the total score for identifying relationships between different variables. Moreover, as suggested by Byrne et al. (19), separated factor scores allow for a better exploration of the complexity of the topic.

Although the multifactorial nature of AWSA is demonstrated, each factor has a different level of importance in the total scale. Our findings indicate that Factor PD dominates in the total scale. Efforts to update this scale to provide a more contemporary measure of attitudes toward women should emphasize improving Factor ED and Factor BD. More items that reflect the equality and BDs should complement the AWSA scale, and items related to sexual activity should be among the first put on the agenda of discussions. Byrne et al. (19) also suggest complementing the AWS scale with issues related to women’ reproductive health.

This study has several limitations. One is related to the sampling methodology. All Bolivian adolescents attended schools in the city of Cochabamba, and all Ecuadorian students attended schools in the city of Cuenca. It is possible that adolescents in other cities or in rural areas would have different opinions. Additionally, the retest of the scale was not performed during our survey. The Cronbach α of Factor ED and Factor BD were rather low (0.48 and 0.24, respectively). As the Spearman-Brown predictive formula estimated a reliability coefficient of 0.73 and 0.60, respectively if a 12-items-length scale would be applied, this indicates the need to add other items to the instrument. Moreover, even if the most important RMSEA index derived from the CFA of the AWSA showed good model fit, other indices (TLI and CFI) did not reach the targeted value (0.95) of good model fit. These limitations indicate the need for additional research to evaluate the factorial validity and reliability of the scale with different populations and in varied contexts, including a test–retest component.

Regardless of these limitations, our sample size was large enough to make comparisons between countries and sexes. The scale itself was quite consistent and reliable both internally and externally.

## Conclusions

This study used AWSA for the first time in Bolivia and Ecuador. It revealed the scale's three-factorial structure and that this structure has a better correlation with different aspects of Bolivian and Ecuadorian adolescent sexual behaviour and experiences than the total AWSA score.

This is especially notable considering the need for instruments that can measure gender attitudes in replicable ways. Taking into account the ease of survey administration and that more egalitarian gender attitudes might have a positive impact on boys’ and girls’ sexual and reproductive health, the three-factor solution of AWSA could have great potential in future research on adolescent sexuality and gender attitudes.
